# Can Bayesian Theories of Autism Spectrum Disorder Help Improve Clinical Practice?

**DOI:** 10.3389/fpsyt.2016.00107

**Published:** 2016-06-17

**Authors:** Helene Haker, Maya Schneebeli, Klaas Enno Stephan

**Affiliations:** ^1^Translational Neuromodeling Unit (TNU), Institute for Biomedical Engineering, University of Zurich and ETH Zurich, Zurich, Switzerland; ^2^Wellcome Trust Centre for Neuroimaging, University College London, London, UK; ^3^Max Planck Institute for Metabolism Research, Cologne, Germany

**Keywords:** autism spectrum disorder, Asperger syndrome, translational research, diagnostic tests, generative modeling, Bayesian inference, Bayesian models, neuroimaging

## Abstract

Diagnosis and individualized treatment of autism spectrum disorder (ASD) represent major problems for contemporary psychiatry. Tackling these problems requires guidance by a pathophysiological theory. In this paper, we consider recent theories that re-conceptualize ASD from a “Bayesian brain” perspective, which posit that the core abnormality of ASD resides in perceptual aberrations due to a disbalance in the precision of prediction errors (sensory noise) relative to the precision of predictions (prior beliefs). This results in percepts that are dominated by sensory inputs and less guided by top-down regularization and shifts the perceptual focus to detailed aspects of the environment with difficulties in extracting meaning. While these Bayesian theories have inspired ongoing empirical studies, their clinical implications have not yet been carved out. Here, we consider how this Bayesian perspective on disease mechanisms in ASD might contribute to improving clinical care for affected individuals. Specifically, we describe a computational strategy, based on generative (e.g., hierarchical Bayesian) models of behavioral and functional neuroimaging data, for establishing diagnostic tests. These tests could provide estimates of specific cognitive processes underlying ASD and delineate pathophysiological mechanisms with concrete treatment targets. Written with a clinical audience in mind, this article outlines how the development of computational diagnostics applicable to behavioral and functional neuroimaging data in routine clinical practice could not only fundamentally alter our concept of ASD but eventually also transform the clinical management of this disorder.

## Introduction

An important precondition for successful translation of basic scientific theories into clinical applications is the knowledge of the most pressing unresolved problems in clinical practice. The care for affected individuals can only be improved effectively if these priority problems are identified and used to guide the design of scientific studies. In heterogeneous disorders, such as autism spectrum disorder (ASD), cross-sectional comparisons of patients vs. controls may provide coarse contours of some characteristics of the spectrum, but are usually not sufficient to inform changes in clinical practice (1).

In this article, we adopt the clinician’s perspective as starting point for outlining how a computational modeling strategy, based on Bayesian theories of ASD (2–4), could inform the development of diagnostic and predictive tests for improving clinical care for individuals with ASD. For the non-clinical reader, we begin with an introduction to the nosology and current clinical management of ASD. For the clinical audience, later sections on computational theories are written in a non-mathematical way and complemented by figures that illustrate basic principles of Bayesian theories.

### Features of ASD – A Brief Overview

#### Nosology

Autism spectrum disorders are developmental disorders of variable severity and heterogeneous phenotypes. Core diagnostic criteria are persistent deficits in social communication and interaction and restricted, repetitive behaviors and interests. Most affected individuals also show altered reactivity to sensory input or unusual interests in sensory aspects of the environment (5, 6) and motor skill deficits or clumsiness (7). The features are present across the life span, but may remain hidden until unmasked by enhanced social demands during development; conversely, they may become less visible in adulthood due to the development of coping strategies. The spectrum ranges from very severe forms – individuals with absent development of verbal language and complete dependence on support – to light expressions of autistic traits that may be masked by learned coping strategies. Generally, there is a smooth transition from pervasive expressions of autistic traits, which cause significant disability and distress to the affected individual, to autistic personality traits that can be regarded as “normal” variations of human personality and do not cause suffering or impairment.

The severe manifestation of ASD, early childhood autism, often co-occurs with intellectual disability and was first described by Leo Kanner in 1943 (8). The term “autism” was introduced to diagnostic classifications in 1976 (ninth revision of the International Classification of Diseases, ICD-9) and 1980 (third revision of the Diagnostic and Statistical Manual of Mental Disorders, DSM-III), respectively. Lighter manifestations were first described by Hans Asperger (9) and introduced to the diagnostic classifications in 1992 (ICD-10) and 1994 (DSM-IV), respectively, as “Asperger syndrome” (10). In contrast to early childhood autism, Asperger syndrome lacks a general retardation in language and is not associated with intellectual disability. Its recognition and introduction to disease classifications triggered a reframing of the earlier category “autism” as “childhood autism.”

This historical background explains why, for almost two decades, the psychiatrists’ and the public’s concept of “autism” was shaped by the severe form. Awareness for lighter manifestations on the spectrum started to grow only slowly after the release of ICD-10 and DSM-IV. Over time, childhood autism and Asperger syndrome were understood as differential expressions on a spectrum with hypothesized similar pathophysiological mechanisms. Accordingly, the latest revision of the DSM (DSM-5) merged them into a single diagnostic category called ASD.

#### Epidemiology

Interestingly, the prevalence of autistic spectrum disorder has substantially increased between 1990 and 2010, and around 1% of the population is now thought to be affected by ASD (11). This rise can be explained by the expansion of the diagnostic criteria (inclusion of Asperger syndrome) and increased awareness of both the public and professionals, leading to more diagnoses without an increased rate of the disorder *per se* (12, 13).

#### Etiology

With regard to etiology, epidemiological studies have long pointed to high heritability and a strong genetic contribution to the risk of ASD, finding concordance rates of 60–70% in monozygotic twins and 18–33% in siblings (14, 15). This strong genetic influence has been further elucidated by recent genome-wide analyses of large populations that suggested two different types of genetic contributions to the risk of ASD (16): while some rare *de novo* mutations can be sufficient to convey risk, in other cases, a wide range (>1000) of common single nucleotide variants may interact in conveying the risk for developing ASD (17). Many of the risk genes for ASD, identified so far, appear to impact primarily on synaptic plasticity and alter connectivity of neural circuits (16). This focus on synaptic connectivity is of relevance for the computational theories discussed below; by contrast, it has not yet been translated into specific therapies.

#### Treatment

Current treatment concepts of ASD include behavioral interventions, psychotherapy, and pharmacological approaches. In children, early behavioral interventions that foster social interaction and speech development (18, 19) are well established and have proven efficacy (20). Adolescents profit from explicit teaching of social skills in groups (21). For adults with ASD, we lack established disorder-specific psychotherapy concepts, so far, that go beyond social skills trainings (22, 23). Available approaches can be divided into psychoeducation (i.e., providing a concept of the disorder and how the individual symptoms relate to it), teaching of coping strategies, and therapy of comorbidities (e.g., depression or anxiety) with currently available options of psychopharmacology and/or psychotherapy. In pharmacotherapy of ASD, the most frequently prescribed and only FDA-approved substance is the dopamine D2 receptor antagonist risperidone (24). This drug has approval for sedation in the presence of aggression or irritability, in ASD. Off-label use of pharmacotherapy mainly rests on the dopaminergic and noradrenergic stimulant, methylphenidate (25). Its effectiveness is mainly documented in the context of comorbid attention-deficit symptoms (26, 27).

#### Theories

Theories of ASD have either focused on the social symptoms of ASD [e.g., as a deficit of theory of mind (28), reduced social salience (29, 30), or a lack of social motivation (29, 30)] or on peculiarities of autistic perception [e.g., “weak central coherence” (31–33)]. By contrast, there is no universally accepted mechanistic theory so far, which provides a unifying explanation across the entire range of autistic symptomatology.

A candidate theory that might fill this gap is what one might refer to as the “Bayesian brain” perspective on ASD (2–4). This is an umbrella term for several similar theories that conceptualize ASD under a predictive coding or hierarchical inference framework and explain autistic cognition as the consequence of fundamental abnormalities in perception and learning. This computational view on ASD suggests concrete models that can be tested by cognitive and neurophysiological studies and that may provide a fundament for developing clinically useful tests. This is the topic of this paper.

### Contemporary Challenges in Clinical Care for ASD

The present clinical management of ASD is not satisfactory in several regards. In the following, we outline some key challenges in diagnosis and treatment of children and adults along the autism spectrum, where we see particular opportunities for Bayesian theories to contribute to improvements.

#### Diagnostic Challenges

Today’s diagnostic criteria defined in ICD and DSM and respective diagnostic procedures were derived from Kanner’s and Asperger’s descriptions of the behavior and development of affected young boys (8, 9). Factors that cause heterogeneity in developmental trajectories of affected individuals and, therefore, observable manifestations of ASD are the degree of severity, the absence or presence of spoken language, gender, age, intelligence, and the individual history of life experience and learning (spontaneous or fostered by training). This heterogeneity causes problems in the diagnosis of ASD.

Since mechanistic definitions and measures of ASD are lacking, diagnosis rests, as for all psychiatric disorders, on symptoms and signs and the developmental history. The Autism Diagnostic Observation Schedule (ADOS) has been developed as a semi-structured assessment tool to standardize clinical examination of the diagnostic criteria of ICD-10 and DSM-IV (34). In combination with the Autism Diagnostic Interview (revised version, ADI-R) (35), which is conducted with parents or caregivers of affected children, it is regarded as gold standard of ASD diagnosis, particularly for children at the more severe end of the spectrum (36). This, however, directly leads us to the first clinical challenge: (*i*) ADOS and ADI-R are time-consuming procedures, which rely on the availability of specifically trained and experienced clinicians. It would be extremely desirable to have a quicker, easier, and less resource-demanding diagnostic test, which could be applied by non-specialized professionals. This would considerably facilitate early diagnosis, which, in turn, is essential for the success of therapeutic (behavioral) interventions at an early stage.

Autism Diagnostic Observation Schedule and ADI-R have less sensitivity in children and adults with higher functioning and milder forms of ASD. This is a result of the greater variance in observable symptoms in these individuals, e.g., due to acquisition of coping strategies (37). In individuals at the lighter end of the spectrum, symptoms may be covered in childhood, until social demands exceed available coping strategies. Later in development, symptoms may become masked by acquired strategies that facilitate social interaction and communication. This variability renders the diagnosis of children, and particularly adults, at the milder end of the spectrum, challenging. Even if these highly functional individuals may show few classical autistic symptoms at first sight, their ability to cope with complex environments and daily demands can be frail. This causes significant exhaustion and suffering, and promotes comorbidities, e.g., depression, anxiety, or substance abuse (38).

The diagnosis of individuals at the lighter end of the spectrum, therefore, requires the detection of subtle signs. For example, peculiarities in social interaction and communication become apparent only in deeper interactions and/or over longer periods of observation. The repetitive nature of behavior manifests itself on larger temporal or spatial scales than in children. Reliable diagnosis often requires an extensive exploration of the patient’s way of perceiving and understanding the world, themselves, and others. Such diagnostic exploration can be instructive in adult ASD patients with a high degree of socioemotional development, who have established a concept of their differences to others. However, compared to non-developmental psychiatric disorders, two complications frequently arise. First, for the patient, his/her autistic symptoms have always been present, and there is no non-affected state to which a comparison could be established. Second, the establishment of abstract representations of the (autistic) self and (non-autistic) others is a core problem in ASD and renders the recognition and description of one’s own particularity difficult.

Another difficulty of recognizing ASD arises in this same group of less severely affected individuals by the fact that, at first clinical contact, their autistic symptoms are often overshadowed by acute exacerbation of secondary effects or comorbidities, such as depression, which often represent their main motivation for seeking clinical help (39–41).

Taken together, a second challenge is the (*ii*) diagnosis of mild forms of ASD due to the great variance of presented symptoms and the difficulty in exploring the inner world of an autistic mind in the absence of quantitative tools. Experienced clinicians, who are able to detect these mild forms of ASD by clinical examination, are even rarer than experts in ADOS/ADI-R. This is because awareness of the lighter forms of ASD has grown only slowly, especially in adult psychiatry, where many older patients remain misdiagnosed because they entered clinical care before the introduction of the diagnostic classification of Asperger syndrome in the 1990s (42, 43).

A third challenge is the (*iii*) detection of very young children at risk, such as siblings of already diagnosed children. Their genetically increased risk for ASD makes them candidates for screening and early intervention in order to optimize their long-term outcome (15). The diagnosis in these very young infant siblings is based on close monitoring of behavioral development (44), but complicated by various onset patterns and the limited repertoire of observable behavior at this early developmental stage (45). Eye tracking of visual scanning patterns is a potentially promising marker of altered cognition at this early stage (46), but remains to be validated in prospective studies.

A fourth diagnostic challenge concerns (*iv*) the assessment of intelligence in ASD patients without spoken language at the severe end of the spectrum. There is evidence that the degree of intellectual disability in ASD individuals with no verbal communication skills is overestimated (47), with possibly severe consequences for the patient. Again, objective and quantitative assessment tools are lacking so far.

In summary, so far, appropriate diagnostic procedures are available only for a limited group of ASD patients with specific degrees of severity and age. Furthermore, their reliance on specific expertise and training makes it difficult for the average psychiatrist to achieve reliable clinical diagnoses of ASD.

#### Treatment Challenges – Behavioral Therapy and Psychotherapy

As described above, several effective concepts of early behavioral intervention and social skills training are established for children across the whole spectrum. By contrast, the follow-up treatment in adulthood still poses considerable problems. This brings us to further concrete challenges:

(*v*) So far, there are no concepts of behavioral interventions that foster socioemotional development of severely affected individuals in adulthood, especially not for those without spoken language and possibly underestimated intelligence (48).

(*vi*) For the mild end of the spectrum, some first concepts of social training for adults do exist (23, 49, 50). However, treatment concepts focusing on “hidden” autistic symptoms in adults, such as sensory oversensitivity, detail-dominated perception, or the need for structure and rituals in self-organization are still to be developed (51).

(*vii*) There is a lack of concepts how the psychotherapy of comorbid disorders, such as depression, needs to be adjusted in the specific context of ASD (52).

#### Treatment Challenges – Pharmacotherapy

(*viii*) There is a complete lack of pharmacological therapies that are motivated by concrete pathophysiological theories and influence either the neurodevelopment in children or tackle the mechanisms behind autistic symptoms in adolescents and adults (53, 54). This lack is remarkable, given that ASD is now considered to represent one of the most strongly heritable and, therefore, biologically determined psychiatric disorders (11, 55).

(*ix*) Trial and error psychopharmacological approaches show some beneficial effect in individual patients. An individualized prediction of treatment response could save time and prevent patients’ suffering from unnecessary side effects of ineffective medication attempts.

## A Computational Framework for ASD

### Computational Approaches to Psychiatric Disorders

Addressing the clinical challenges highlighted above represents a daunting problem. Without a fundamental mechanistic explanation for the manifold clinical manifestations of ASD, we lack a fundament for developing diagnostic tests and new treatment strategies. Clearly, this situation is not unique to ASD: psychiatry generally lacks mechanistically grounded diagnostic tests. In contrast to other areas of medicine where hidden disease mechanisms can often be inferred by advanced measurements of downstream consequences (e.g., biochemical or immunological assays of blood samples), the diagnosis of psychiatric disorders is hampered by lack of access to disease-relevant tissue (i.e., the brain) and the absence of biochemical or genetic markers with predictive utility (56, 57). Similarly, while structural neuroimaging techniques are used in clinical practice to rule out non-psychiatric disorders (“organic” causes), their functional counterparts are remote from neuronal processes of interest, e.g., neuromodulatory signals. More than two decades of functional neuroimaging research have yielded no application that has entered routine psychiatric practice so far (1, 58).

**Figure 1:**
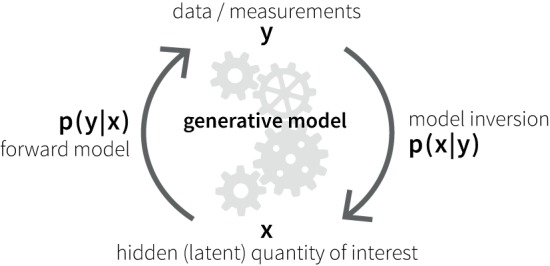
**Schematic of the principles of a generative model**.

A potential alternative to classical neuroimaging is offered by emerging computational methods based on generative models of measurable behavior or brain activity (59). Generative models are forward models that describe how latent (hidden) cognitive or physiological processes *x* could have generated experimentally measured data *y* (Figure 1). Based on Bayes’ theorem (60), generative models allow for solving the inverse problem of inferring the hidden processes from empirical data, yielding the posterior probability *p*(*x*|*y*) of the hidden cause of interest. This computational approach allows one to compute subject-specific parameters that determine the hidden neuronal or cognitive states of a circuit. Furthermore, the plausibility of different generative models can be evaluated using statistical model comparison techniques (61, 62).

In the context of psychiatric disorders, the relatively straightforward availability of behavioral or brain activity measurements suggests that validated generative models could be developed into clinically applicable “computational assays,” in analogy to biochemical assays in internal medicine (63). A series of recent proof of concept studies (64–66) have been an important stimulant for the development of the emerging field of computational psychiatry (59, 67–69).

### The “Bayesian Brain”

#### Overall View

**Figure 2:**
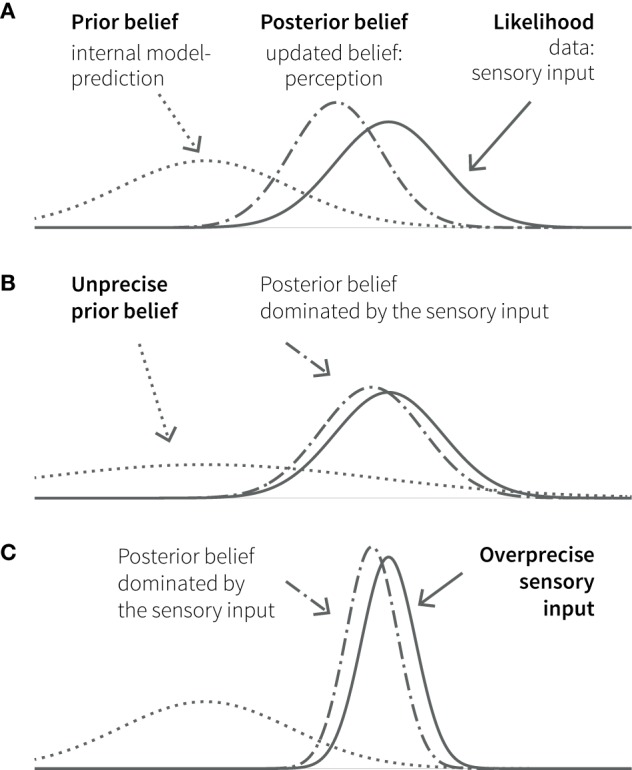
**Principles of Bayesian inference**. **(A)** A prior belief (knowledge, expectation, or prediction; dotted line) is combined with the likelihood (observed data, e.g., sensory input; solid line) in the form of Gaussian probability distributions. The width of the curves represents uncertainty (variance); its inverse (the narrowness of the curve) represents the precision of or the confidence in the respective belief or data. The resulting posterior belief (dashed-dotted line) represents the updated belief, as a precision-weighted compromise between prior and likelihood, which is dominated by the quantity with higher precision. In cognition, perception can be understood as the formation of a posterior belief in response to sensory input. The lower panels show two additional situations, in which the posterior (perception) is biased toward the (sensory) data: in one case because the prior (belief) is unprecise **(B)**; in the other, because the (sensory) data are over-precise **(C)**.

Bayesian inference is remarkably analogous to perception, where the challenge is to distil meaning from noisy and ambiguous sensory inputs. Based on the principles of probability theory, Bayesian interpretations of cognition refer to “beliefs” as probability distributions (i.e., a probabilistic representation of a particular state of the world) and how these beliefs are updated in the light of experience (observed data). Bayes’ theorem describes how the observation of new data (likelihood) changes a prior belief into a posterior belief. This posterior belief represents the inference about the most likely cause behind the observed data, given the previous knowledge, and becomes the new prior belief or prediction for future observations (see Figure 2).

We refer to a Bayesian perspective on cognition as the “Bayesian brain hypothesis,” an umbrella term for several related concepts (70–73). All of them regard the brain as an inference machine, resting on a generative model of sensory inputs, which are caused by states of the environment. For simplicity, we will often refer to this generative model of sensory inputs as the brain’s internal model of the external world. By inverting its generative model, the brain can infer the most likely environmental state (cause), given the sensory inputs it has received. Furthermore, the brain can use its internal model for prediction and compute the probability of certain environmental states arising from chosen actions (74).

This Bayesian interpretation of perception has become a widely used perspective and has enabled the understanding of many perceptual phenomena, including a unification of perceptual laws (75), multisensory integration (76, 77), and the nature of sensory illusions (78).

#### Learning

**Figure 3:**
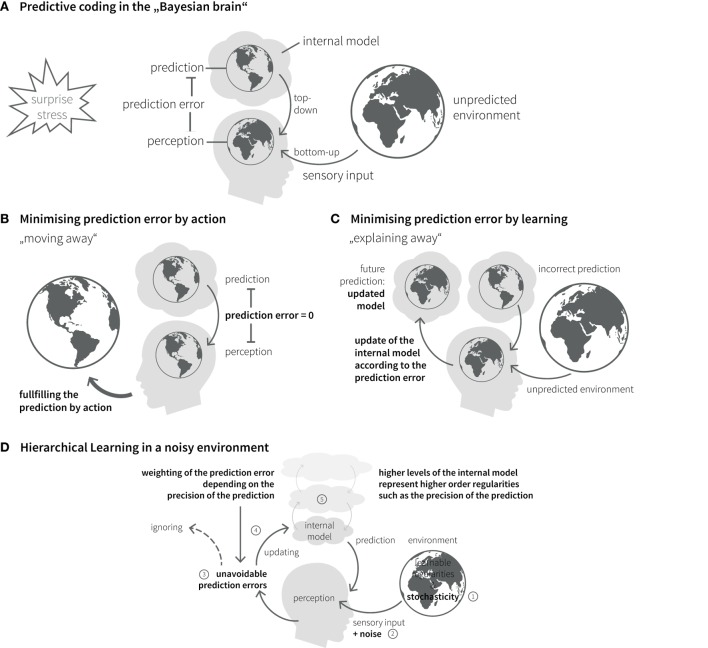
**Bayesian inference in the brain**. **(A)** The “Bayesian brain” predicts (based on its internal model) the incoming sensory input from the environment and compares it with the actual input. The difference between prediction and sensory input is called prediction error. The brain’s homeostatic goal is to minimize prediction errors. Prediction errors can be reduced in two ways: action or learning. **(B)** Predictions can be fulfilled by choosing actions that lead to expected sensory inputs. **(C)** Incorrect predictions can be adapted according to prediction error. Under this model update (learning), the prediction error is explained away. **(D)** Due to stochasticity in the environment (1) and noise of sensory channels (2), prediction errors can usually not be explained away completely (3). Their impact on belief updates depends on the relative precision of sensory input and prediction (4), which is coded in higher levels of the internal model (5).

The brain’s internal model can be updated over time; this corresponds to learning and rests on a key quantity, the prediction error. This is the difference between the predicted and the actual sensory input and constitutes part of an approximation to surprise (Figure 3A) (72). An influential recent hypothesis – the so-called “free-energy principle” (79, 80) – is that perception and action selection are governed by one overarching objective: the minimization of surprise and hence the avoidance of prediction errors. The free-energy principle essentially views the Bayesian brain as implementing a homeostatic principle of information processing where the absence of prediction error represents the set point against which actions are chosen.

In principle, there are two ways of minimizing prediction errors. First, a prediction might be fulfilled by choosing the appropriate action. This includes moving one’s sensors (e.g., eyes, limbs, or the entire body) to parts of the environment where the sensory inputs better match the predictions (Figure 3B). Second, the brain can use surprise as teaching signal to adjust its beliefs. This corresponds to learning or updating its generative model, so that the current prediction error is explained away and more accurate future predictions become possible (Figure 3C).

#### Uncertainty

Importantly, however, not all unpredicted inputs are equally informative. Due to stochasticity in the environment and noise inherent to all sensory organs, not all prediction errors signal true changes in learnable regularities. Given this uncertainty, updating the generative model in response to each and every input could result in overfitting, i.e., an overly precise and brittle model with limited generalizability over time. Instead, belief updates should be governed by the balance between two quantities: the uncertainty about the sensory input (i.e., expected signal-to-noise ratio), and the uncertainty of the prior belief. For a wide range of learning models, this can be described by an iconic equation (Eq. 1). That is, any change in belief is proportional to prediction error, but weighted by the ratio of the precision of the sensory input and the precision of the prior belief (81).

(1) $$\Delta \text{belief}\propto \frac{{\text{precision}}_{\text{input}}}{{\text{precision}}_{\text{prior belief}}}\times \text{prediction error}$$

This precision ratio can be regarded as dynamic learning rate: it is high whenever the confidence in the sensory input (bottom-up information) is higher than the confidence in the current belief (top-down predictions of the model), or conversely, when the uncertainty of the predictions provided by the internal model is higher than the uncertainty about the sensory input. The higher this precision ratio, the more informative surprising input and the more pronounced the updating of the internal model.

#### Cognitive Hierarchies

The causal structure of the world, with its nested spatial and temporal scales, implies that the brain’s internal model also possesses a hierarchical structure, which is a natural form for Bayesian inference: hierarchical models allow to encode information about the precision of beliefs at one level by values of hierarchically higher levels (81–83) (Figure 3D). In a hierarchical setting, information passes from sensory cortical areas to update higher levels within the cortical hierarchy, representing more and more abstract information on higher temporal and spatial scales [cf. (84)]. The more precise these abstract representations are established, the less impact any surprising experience has on revising the established internal model. In other words, more precise high-level beliefs exert stronger guidance in interpreting new experiences and shield against continuous reshaping of the brain’s model of the external world.

#### Homeostasis and Psychopathology

Theories like predictive coding or the free-energy principle are theories of cognitive homeostasis: they describe how a system responds adaptively to a mismatch between desired (predicted) inputs and actual inputs. A mismatch (prediction error, surprise) represents a stressor to the cognitive system and triggers adaptive responses, such as the change of internal settings (update of beliefs) or outputs (motor actions). In the context of predictive coding, the adaptive updating of internal beliefs is also referred to as “explaining away” prediction errors. An acute or chronic impairment in explaining-away prediction errors represents a form of “cognitive stress” and will be registered by higher model levels on the cognitive hierarchy involved in monitoring the cognitive performance of the lower levels of the internal model (85).

Since the majority of current computational concepts of psychiatric disorders regard aberrant learning and inference as core components of maladaptive cognition (59), the three elements in Eq. 1 – prediction, prediction error, and precision – offer an interesting perspective for clinicians. They suggest that cognitive stress due to maladaptive inference arises from alterations in one or several of these three core components. These quantities span a three-dimensional space where different pathologies could be located (86). This means that similar psychopathological phenotypes (based on disturbances of Bayesian inference) could arise by several pathomechanisms, affecting differentially the biological basis of one or more of these computational quantities.

Individual differences in the structure of internal models or model parameters represent specific cognitive styles or cognitive strategies and would manifest in behavioral differences. Provided one has generative models that can infer, from subject-specific behavior, on the structure and parameterization of an individual brain’s generative model, powerful diagnostic tests might become possible. Such computational assays – which correspond to generative models of generative models – would become particularly powerful, if mappings between the above computational quantities and specific neurophysiological entities could be established.

### A “Bayesian Brain” Perspective on ASD

#### A Clinician’s View as Starting Point

Autism spectrum disorder is clinically characterized by prominent perceptual aberrations, which appear to map naturally on impairments of hierarchical Bayesian inference. Individuals with ASD have striking difficulties in distinguishing between relevant (informative) details and irrelevant, random changes. For example, during the interaction with another person, a patient with ASD may direct more attention to a new haircut or the color of the shirt than to the emotional expression of the other’s face. Furthermore, ASD patients struggle to establish generalizable, abstract representations by making meaningful connections and tend to have overly precise representations of single observations and detailed sensory aspects. For example, they take expressions too literally, or do not know how to behave in situations that only subtly differ from known constellations; small details, e.g., variations in location or timing, can be sufficient to induce feelings of uncertainty and lack of control. Finally, they experience a chronic sensation of being unprepared for whatever happens, unless they can exert control (and thus avoid surprise) in a stable, well-known environment. This may underlie their desire for fixed rituals, such as never changing the exact order of a sequence of actions in everyday life.

#### Summary of Current Theories

Several recent articles have suggested that aberrant Bayesian inference underlies perceptual abnormalities in ASD. For example, Pellicano and Burr (2) proposed that ASD is characterized by overly flat priors, which lead to percepts dominated by the sensory input. Their proposal was extended by Lawson et al. (3), who pointed out that the precision of top-down predictions need to be weighed against the expected precision of bottom-up sensory input (Figure 2C; compare Eq. 1). They highlighted the importance of postsynaptic gain control as a potential neurobiological mechanism for precision weighting and hypothesized that GABA, acetylcholine, and oxytocin could play a central role in the adjustment of precisions at different hierarchical levels. Finally, Van de Cruys et al. (4) pointed out that normal, or even tight, high-order beliefs could be present in ASD, provided that their effects are outweighed by overly high precision of sensory prediction errors. Again, this speaks to the crucial role of precision ratios (see Eq. 1) for dynamically governing belief updates across hierarchical levels.

#### Autistic Perception

**Figure 4:**
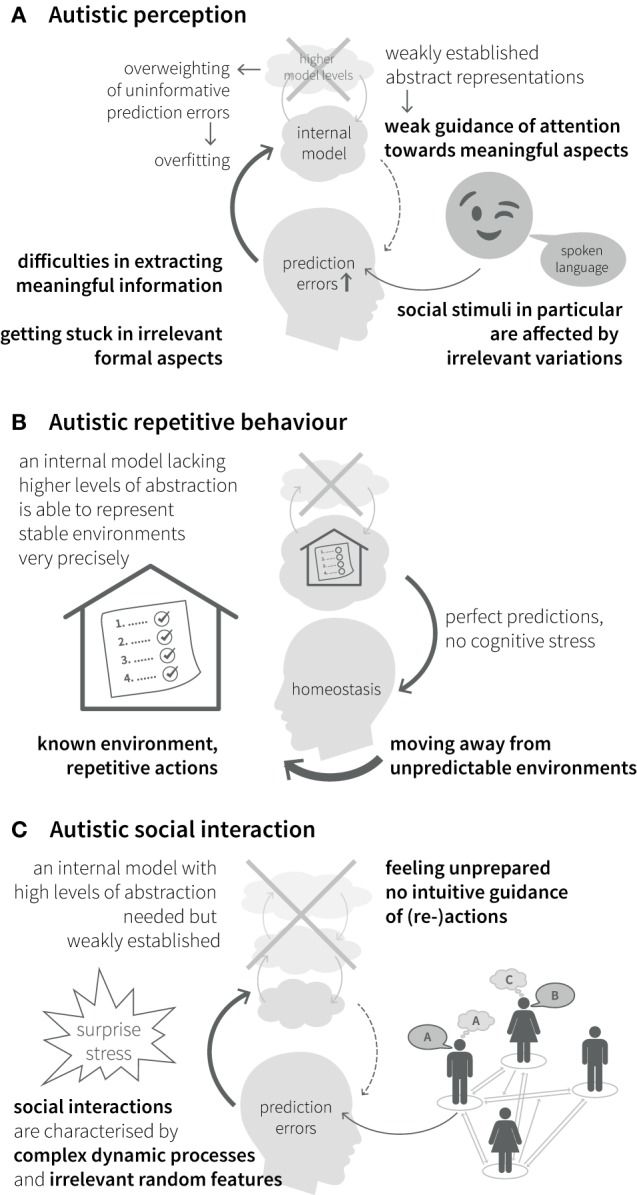
**Autistic symptoms from a Bayesian viewpoint**. **(A)** *Perception*. Weakly established abstract representations provide predictions with low precision and fail in guiding attention toward informative stimuli. Especially for complex stimuli with frequent irrelevant variations, such as social stimuli, attention can be attracted by unpredicted changes in irrelevant formal aspects. Overweighted low-level prediction errors, due to overly high sensory precision, cause overfitting of the internal model and difficulties in extracting meaningful information. This impairs the establishment of high-level (abstract) representations and reduces the ability to explain away future prediction errors. **(B)** *Behavior*. Minimization of prediction errors is achieved more easily by moving away from unpredictable environments into highly regular environments with repetitive actions and rituals, since they can be precisely predicted by a model without many levels of abstraction. **(C)** *Interaction*. Social interactions in particular are characterized by complex dynamic processes and irrelevant random features, which require regularization and suppression by an internal model with a high degree of abstraction and precise predictions. A relative lack of this model makes it difficult to infer the causes of social stimuli (i.e., understand the meaning of social processes) and, thus, to interact with others.

Impairments of hierarchical Bayesian inference provide an explanation for the different clinical symptoms described above. Specifically, aberrant updating of the internal model due to overestimating the precision of bottom-up sensory input in relation to the precision of top-down predictions (see Figures 2B,C and Eq. 1) would lead to a perceptual style, which is dominated by detailed but irrelevant aspects of the environment and a difficulty in establishing stable and precise representations of abstract quantities at high levels of the perceptual hierarchy (Figure 4A). Interestingly, this suggests a concrete computational mechanism for the long-standing concept of “weak central coherence,” which postulates that processing of local details dominates perception in ASD, at the expense of global integration of information (31, 32). The overweighting of uninformative sensory prediction errors leads to constant fluctuations and large uncertainty at higher levels in the generative model, which represent overarching, abstract concepts. Additionally, a relative failure of encoding or updating high-level precision implies that even relatively predictable stimuli will be perceived as continuously surprising. Overall, the proposed dysbalance in low- vs. high-level precisions result in an overfitted model that is dominated by sensory details and has limited generalizability.

#### Autistic (Inter-)Action

Our inner representation of the world not only explains what we perceive but also guides our interactions with the world. Models that concentrate on detailed aspects of the sensory world (i.e., overly high precision of sensory prediction errors) elicit actions that serve to explain away these prediction errors (such as seen in precisely defined autistic rituals, Figure 4B). Importantly, this interferes with adaptive actions in the presence of irrelevant changes in details. This difficulty of establishing and applying abstract representations to interpret sensory inputs and guide action causes greatest difficulties in highly unpredictable environments, such as completely novel situations or social interactions, which are particularly dynamic and ambiguous (noisy) (Figure 4C). Similar problems can be expected in areas, where highly abstract contents are exchanged, like in human communication. Hence, the Bayesian perspective offers an intuitive explanation for the theory of mind deficits in ASD. From this point of view, ASD can be seen as a general disorder of hierarchical inference that manifests most prominently in the social domain without being limited to it.

#### The Spectrum Nature

Autism spectrum disorder shows pronounced heterogeneity, both with respect to the behavioral phenotype and severity of impairments. The hypothesized fundamental mechanism affected in ASD, hierarchical Bayesian learning, is based on three main pillars: predictions, prediction errors, and the respective precisions. As indicated above, these three variables span an explanatory space of the potential computational pathomechanisms that could underlie impairments in hierarchical Bayesian learning. For example, predictions and/or prediction errors could be incorrectly calculated at the level of specific neurons (e.g., abnormal integration of dendritic inputs to supragranular pyramidal cells), or they could be conveyed incorrectly to target neurons (e.g., presynaptic or postsynaptic deficiencies of long-range connections). Alternatively, as suggested by Lawson et al. (3) and Van de Cruys et al. (4), precisions could be tuned abnormally. This could arise, for example, from a dysregulation of neuromodulatory transmitters (e.g., dopamine, acetylcholine, noradrenaline), which affect postsynaptic gain by modulating calcium-dependent potassium channels. Due to the anatomy of their specific projection pathways, the individual neuromodulators (and their potential dysregulation) impact differentially on different cortical areas (e.g., affecting different sensory modalities and different levels of the cognitive hierarchy).

This list of possibilities is not exhaustive, but illustrates how the phenotypic and clinical variability of ASD patients could arise from different impairments of hierarchical Bayesian inference. In other words, different autistic phenotypes could arise from different impairments in the computation of key variables, such as precisions or prediction errors. The present framework, thus, offers a broad range of explanations, how the spectral nature of ASD – in severity and phenotype – could arise, and suggests possibilities to disentangle potential mechanistically different subtypes of the disorder.

#### Developmental Trajectories

Bayesian theories of ASD also provide an explanation for the spectrum of developmental trajectories and how they are influenced by the history of life experience and learning. The hypothesis that ASD is characterized by an inflated ratio of the precision of bottom-up sensory input in relation to the precision of top-down predictions (Eq. 1) makes concrete predictions about the learning conditions under which ASD patients can benefit. These conditions match precisely the conditions to which behavioral therapy for children with ASD has converged over the years (20): a well-known environment that causes little surprise and offers new inputs with little noise (unexplainable variability) across many repetitions. According to the therapeutic experience of the authors, new actions are best learned by step-by-step instruction (as opposed to mere observation), and abstract concepts are easiest learned through explicit definition (rather than by intuitive buildup) (21). If such conditions are present and an affected individual is exposed to enough inputs over a longer time, a sufficiently rich representation of the world and successful behavioral strategies can be learned. Variations in the occurrence of these conditions in different domains or stages of life could explain the evolution of symptoms over time in form of developmental steps and functional adjustment through training.

#### Comorbidities

Comorbidities occur frequently and can impede this progress of adjustment. The most frequent and relevant ones are stress-related disorders, such as depression or anxiety (87). From the Bayesian brain perspective, these can be interpreted as a consequence of chronically elevated cognitive stress levels in individuals with ASD, which originates from their impaired ability to explain away prediction errors. As alluded to above, a persistent impairment of minimizing prediction errors in a specific processing stream is likely registered by higher systems for self-monitoring (85) and creates the continuing experience of an unpredictable environment of bewildering complexity. This may engender to the meta-cognitive evaluation that the brain’s model of the world is inadequate to deal with its complexity, leading to estimates of low self-efficacy (88) and a pervasive feeling of vulnerability (Stephan et al., In preparation^1^). This, in turn, may constitute a fundament for the development of comorbidities like depression and anxiety in ASD.

#### Similarities with Schizophrenia

It is of interest to note that similar Bayesian theories have previously been stated in the context of schizophrenia (63, 68, 89–92). ASD and schizophrenia exhibit some striking similarities in certain symptoms. In fact, the term “autism” was introduced by Bleuler when referring to symptoms like social withdrawal in schizophrenia (93). Perceptual aberrations, e.g., a reduced tendency to illusions (75), take a similar form in both disorders, suggesting that similar impairments in Bayesian inference may be present in both disorders. However, the markedly different age of onset of the two disorders requires an explanation why a common mechanism, such as aberrant belief precisions, may reach a critical threshold at different times during development. One might speculate that this results from epigenetic differences in the two disorders, i.e., differential interactions of genetic predispositions with environmental influences. Also, salient symptoms as the presence of positive symptoms in schizophrenia deserve attention (94). Notably, psychotic episodes are not uncommon in adolescents with ASD, although they are mostly shorter in duration and present less well-established delusions than in schizophrenia (10, 38, 95). Furthermore, neurobiologically, ASD and schizophrenia share putative risk genes (96) and may involve analogous abnormalities in the neuromodulatory regulation of postsynaptic gain, as described further below (3, 92).

## Considerations for Future Studies

### Present Empirical Findings

The recently developed Bayesian brain theories of ASD can not only explain cardinal features of autistic symptomatology but also a broad range of previous empirical findings in the domain of neuropsychology, neurophysiology, and functional neuroimaging. A comprehensive overview of these interpretations goes beyond the scope of this article, but can be found in several recent articles on Bayesian brain theories of ASD (2–4).

In the elaboration of the Bayesian brain hypothesis of ASD (2) and subsequent theoretical papers (3, 4, 97–99), results from numerous earlier cognitive and psychophysical studies were re-interpreted *post hoc* in the novel framework. So far, only a few subsequent experimental studies in individuals with ASD were designed *a priori* to test the predictions of the Bayesian brain hypothesis. For example, a psychophysical study by Palmer et al. (100) showed that adults with ASD exhibited the typical perceptual effects of the rubber hand illusion but showed reduced influence of the illusion on subsequent grasp movements. This result speaks against a general insusceptibility of ASD individuals to the illusion and is better explained by a stronger weighting (precision) of proprioceptive sensory input relative to reliance on prior context information. Another psychophysical study demonstrated decreased loudness adaptation in adults with ASD, in accordance with the notion that a failure of updating precision of beliefs (predictions) slows down surprise reduction during a series of predictable stimuli (101). Similarly, an EEG study using a mismatch negativity design which showed that, compared to unaffected participants, children with ASD display a diminished top-down P300 amplitude for unexpected stimuli and a greater amplitude for expected stimuli. Again, both results are compatible with a reduction in the precision of predictions (priors) in autistic patients compared to healthy subjects (102). Finally, a behavioral study of adults with ASD showed reduced learning performance in a volatile compared to a stable environment, consistent with the proposed inability to establish stable high-level representations of abstract rules (103).

These emerging empirical findings speak to the utility of the Bayesian brain perspective for understanding aberrant computation and pathophysiology in ASD. However, a direct link to clinical challenges and practice has been missing so far.

### Modeling Cognition

Bayesian brain theories can be implemented by a variety of different models (72, 104). These typically take a hierarchical form where messages are exchanged bottom-up and top-down between layers, resembling the architecture of the cortex with its hierarchically organized connections (105). One well-known hierarchical Bayesian model is predictive coding (72, 106), which posits that each information processing level (e.g., cortical area) predicts the activity in the next lower level of the hierarchy and sends this prediction via top-down or backward connections. The lower level computes a prediction error (the difference between its actual and the predicted activity), weighted by the precision of the prediction, and returns this precision-weighted prediction error by bottom-up or forward connections to the higher level where it serves to update the prediction. This process takes place across all hierarchical levels until prediction errors are minimized throughout the entire hierarchy.

This model, and variants thereof, has already found widespread application to other psychiatric disorders with perceptual aberrations, in particular schizophrenia (63, 89, 90, 92, 107). Notably, it contains the three main building blocks of inference mentioned above (predictions, prediction errors, and precision), each of which has some putative physiological counterparts. For example, prediction error signaling in cortex likely rests on fast glutamatergic transmission, probably involving fast AMPA receptors under regulation by slower NMDA receptors (91, 108); predictions are probably conveyed by glutamatergic backward connections and exclusively via slow NMDA receptors (72, 91); finally, precision, which is essential for the context-dependent weighting of prediction errors, may be regulated by neuromodulators (dopamine, acetylcholine, noradrenalin, etc.) and local GABAergic interneuron activity, both of which modulate the gain of the postsynaptic neuron (92).

Predictive coding is a model of inference and does not directly account for across-trial learning. A hierarchical Bayesian model that shares key features with predictive coding but focuses on learning under the influence of different forms of uncertainty is the Hierarchical Gaussian Filter (HGF) (81, 109). Using a variational approximation, it derives analytical update equations with subject-specific parameters that encode an individual’s approximation to ideal Bayesian learning. The HGF can be applied in a meta-Bayesian way, with an examiner (e.g., psychiatrist) using Bayesian inference, to infer on Bayesian inference processes that underlie the observed behavior of a patient (110).

The HGF is particularly suitable for complex probabilistic learning tasks, whose statistical structure is volatile. Its hierarchical structure captures the relations of coupled quantities in the world, such as how sensory inputs depend on probabilistic associations (contingencies) which, in turn, evolve as a function of environmental volatility. Each of these quantities evolves as a Gaussian random walk, with its precision determined by the level above, and belief updates are governed by precision-weighted prediction errors as shown by Eq. 1. This formulation has found successful application in several recent studies with healthy participants, showing that the HGF explains learning and decision-making under volatility better than other commonly used models (111–113). Associative learning tasks that include phases of volatility (i.e., weakening or reversal of previously learned associations) represent attractive paradigms to study potential peculiarities in hierarchical inference in individuals with ASD, since their problems in establishing abstract high-level representations arise mainly in contexts with either high levels of sensory noise (where increased precision of bottom-up signaling is detrimental) or temporal uncertainty (where weak top-down predictions are further diminished by volatility).

### Modeling Neurophysiology

The Bayesian brain perspective is an attractive framework for understanding pathophysiology in ASD. In principle, one could imagine that carefully designed behavioral tasks alone could support model-based diagnostics and predictions. However, the utility of the Bayesian brain perspective extends beyond modeling cognition. In particular, generative models of behavior can finesse analyses of functional neuroimaging data and allow to identify potential neurophysiological fundaments of computational processes. For example, trial-by-trial estimates of computational quantities, such as prediction errors or precisions, can be used as parametric modulators in a general linear model (GLM) of fMRI data, an approach commonly referred to as “model-based fMRI” (114). This approach has been used, for example, to identify links between activity in neuromodulatory nuclei and computational trajectories, such as (precision-weighted) prediction errors or uncertainty (111, 115–117). For example, Iglesias et al. (111) showed that low-level precision-weighted prediction errors about visual stimulus outcome were reflected by fMRI activity in the dopaminergic midbrain, whereas high-level prediction errors about stimulus probabilities were encoded in the cholinergic basal forebrain.

Establishing computational neuroimaging probes of neuromodulation is a theme of general importance in computational psychiatry, since this may provide a physiologically interpretable stratification of patients from spectrum disorders with direct implications for individual treatment (118). In the context of ASD, a dysregulation of neuromodulatory mechanisms could underlie abnormal precision-weighting of prediction errors (and the ensuing behavioral consequences) in a subgroup of patients. While group-level abnormalities of serotonergic and dopaminergic transporter activity have been reported by previous studies using single-photon emission computed tomography and positron emission tomography in individuals with ASD (119, 120), the results are mixed, presumably due to the spectrum nature of ASD. This likely pathophysiological heterogeneity is also reflected by the highly variable response of ASD patients to a variety of commonly used psychiatric drugs, which affect neuromodulatory transmitters, including antipsychotics and stimulants (121, 122). Individualizing pharmacotherapy would require a non-invasive and easily applicable assay of neuromodulatory function in individual patients. Developing such assays on the basis of generative models of behavior and computational functional neuroimaging represents a central goal for model-based diagnostics in ASD.

In addition to model-based fMRI investigations that are supported by modeling of behavior, the Bayesian brain perspective on ASD also makes predictions about neurophysiology that can be examined on their own, without reference to behavior. Most importantly, as alluded to above, the form of hierarchical Bayesian models such as predictive coding, with their emphasis on exchange of predictions and prediction errors across hierarchical levels, shows a remarkable correspondence to structural and functional principles of the cortex (72). That is, sensory processing streams in cortex, such as the visual, auditory, or somatosensory system, are characterized by a hierarchical structure that rests on interregional forward (bottom-up) and backward (top-down) connections with laminar and functional specificity (123–125). Numerous neurophysiological studies have provided evidence for signaling of prediction errors along forward connections and predictions along backward connections [(63, 72) for reviews see (126, 127)]. As a consequence, a putative pathophysiological mechanism that alters the signaling of predictions or prediction errors, respectively – either on their own or by abnormal precision-weighting – should be expressed in selective changes of forward or backward connections in a particular sensory hierarchy. This implies an important role for models that can infer changes in these connections from functional neuroimaging data.

A generative modeling framework, which is capable of differential inferences about forward and backward connections, is dynamic causal modeling (DCM). This approach has been implemented for a range of measurements, including fMRI (128) and EEG (129). DCM of fMRI represents a generative model of local BOLD signals in a distributed set of regions, describing how the measured fMRI signals arise from net population activity of large populations of neurons that communicate via synaptic connections (128). Inverting this model allows for estimating the strengths of directed synaptic connections between regions, thus moving beyond purely correlational statements about network architecture as obtained by functional connectivity analyses.

Dynamic causal modeling is beginning to find widespread use in psychiatry. In the context of schizophrenia, several studies have been conducted in individuals at risk (130, 131) and in patients during the first episode (132), early course (133), or chronic state of schizophrenia (134). These studies demonstrated differences in functional network architecture and effective connectivity compared to healthy controls across various tasks. More recently, DCM studies have been conducted in individuals with ASD. Radulescu et al. (135) examined connectivity during a verbal fluency task and found that adults with ASD relied more strongly on bottom-up connections, compared to dominance of top-down connections in the control group. Gu et al. (136) used DCM to infer connection strengths between the extrastriate body area, the anterior insular cortex, and the lateral prefrontal cortex during an empathy for pain task. They found a greater disinhibition in the anterior insula in a group of high-functioning adults with ASD compared to a control group. In brief, the results of DCM studies in both schizophrenia and the ASD point to dysconnectivity of cortical areas and altered functional integration at different levels of perceptual hierarchies.

Dynamic causal modeling also allows for incorporating trial-wise computational quantities obtained from generative models applied to behavioral task data (e.g., precision-weighted prediction errors). This opens new avenues to derive a joint physiological–computational characterization of network dynamics during the performance of a task. Vossel et al. (137), for example, have demonstrated in healthy adults, using DCM for fMRI and the HGF, that during a combined attention/learning task (Posner’s paradigm), the functional coupling between temporal and frontal regions was modulated by trial-wise estimates of attention (precision of the predictability of targets).

The fine temporal resolution of electrophysiological recordings provides much richer information on neurophysiological processes than fMRI. The generative model of DCMs for EEG exploits this information to describe how electrophysiological measurements are generated from cortical microcircuits (columns) with synaptic connections between different types of neurons (129). Validation studies in humans and animals have shown that DCM for EEG is capable of capturing short-term changes in synaptic efficacy, such as neuromodulation of glutamatergic receptor conductances, and distinguish different types of synaptic plasticity in cortical microcircuits (66, 132, 138–140) and, therefore, represents a promising approach to quantify the status of neuromodulatory systems in cortical microcircuits and may provide a foundation for clinically applicable tests.

### Implications for Translational Studies

The pathomechanistic hypotheses of ASD that arise from the Bayesian brain perspective and the computational modeling techniques afford new avenues toward developing clinical tests for addressing problems of diagnosis and treatment in ASD. Initially, this will require a series of translational studies that evaluate the practical utility of different paradigms and models in patient studies.

In a first step, the preliminary evidence for disturbances in Bayesian inference in ASD (100–103) should be extended to multilevel hierarchical Bayesian learning paradigms in individuals with ASD. For this, the HGF framework provides a suitable platform as it allows for obtaining individual parameter estimates (encoding the influence of different forms of uncertainty or precision on learning) from relatively short behavioral measurements. At its simplest, one could adopt a cross-sectional design and test for group differences in these parameter estimates between ASD patients and adequately matched control groups. Additionally, the statistical comparison of different alternative models (within or beyond the HGF framework) could yield information about potential different subgroups of patients applying different cognitive strategies [cf. (113, 141)].

Model comparison also addresses an issue that has been a limitation in neuropsychological assessments of high-functioning individuals with ASD. Many tasks can be solved by applying different cognitive strategies, and this is not necessarily reflected by differences in average performance levels. Such hidden individual differences can be detected by formulating alternative computational models, each reflecting different cognitive strategies, and subjecting them to Bayesian model selection (62). Importantly, this can clarify whether any individual differences in task performance are due to the deployment of different cognitive strategies, or due to differences in implementing these strategies (141).

The diagnostic challenges in ASD particularly affect those individuals whose symptoms deviate from the classical clinical picture described by Kanner and Asperger, due to the factors described in the Section “Introduction.” To test the hypothesis of a shared underlying pathomechanism despite diverse clinical presentations, the behavioral cross-sectional studies described above should be carried out both in children with the classical picture and diagnosed by the current gold standard (ADOS/ADI-R) and in individuals with less specific symptoms but diagnosed with ASD by experienced specialists.

The behavioral studies are ideally combined with the acquisition of functional neuroimaging data. This would enable the deployment of a model-based analysis, using computational trial-wise quantities, such as precision-weighted prediction errors, to test for potential differences in neuromodulatory regions of interest [cf., Ref. (111)]. This investigation could clarify to what degree the impairment of different neuromodulatory systems across patients, causing disturbances in hierarchical inference, could represent a source of heterogeneity in individuals with ASD. Furthermore, one could also inform models of effective connectivity like DCM by trial-wise computational quantities [(137), cf., Ref. (142)], and test for group differences in Bayesian message passing in sensory hierarchies.

Notably, all of the above possibilities could be pursued in a multivariate setting, where individual parameter estimates are used to specify the feature space for subsequent supervised (classification or regression) or unsupervised (clustering) learning. This strategy is known as “generative embedding” (64, 65) and offers two major advantages over conventional machine learning applications that operate directly on features of the measured data. First, provided one has a good model, generative embedding typically results in substantially higher performance since the model is used as a theory-led feature selection device, which retains only dimensions of interest and discards irrelevant data features. Second, the classification or clustering results have a mechanistic interpretation since the dimensions of the feature space are given by specific model parameters. In ASD research, generative embedding based on hierarchical Bayesian models of behavioral data in conjunction with DCMs of effective connectivity might allow for designing powerful classifiers that support differential diagnosis. Additionally, an unsupervised approach would be of interest in order to identify potential mechanistically different subgroups. This is of special interest, since pharmacological therapy in ASD is presently trial and error; furthermore, many pharmacological substances targeting neuromodulatory mechanisms are available that have not yet found therapeutic use in ASD.

If initial studies indicate that discriminative parameters can be obtained and have high predictive power regarding diagnosis and/or treatment response, a necessary next step would be to turn the research-driven paradigms into easily applicable clinical tests. To minimize the influence of motivation and attention – potential limitations to this approach – and to ensure patient compliance and applicability in non-research settings, any cognitive paradigms would have to be relatively short or inherently appealing, with little or no need for specific instructions. Attractive candidate paradigms include tasks that neither require verbal instruction nor voluntary responses, such as implicit learning tasks or games that register involuntary responses such as eye movements (143) or electrophysiological mismatch negativity (144). The former could also be developed for infants (46). This would open up the possibility of characterizing potentially affected infants at a very early stage and following them up in longitudinal studies to study their developmental trajectories.

If modeling results imply that subgroups of patients exhibit pathophysiological mechanisms that can be targeted by existing interventions – for example, disturbances of specific neuromodulatory systems that could be targeted by selective drugs, or abnormal changes in the precision of beliefs about sensory inputs to which psychotherapeutic interventions might be directed – this would provide a foundation for planning randomized clinical trials. This could involve longitudinal clinical studies with pharmacological interventions, optimally in conjunction with tailored psychotherapeutic interventions (145) to guide the learning of new experiences in an optimally designed context in adults or combined with early behavioral training in children.

## Potential Benefits for Future Clinical Practice

Clearly, solving the current clinical problems is an ambitious and long-term goal, which will take many years to reach. However, we believe that the framework outlined above has the potential of addressing the challenges described in the Section “Introduction.”

### Diagnosis

In the long run, clinically applicable, computerized trial-by-trial cognitive paradigms with accompanying generative models for the acquired data and “pushbutton” procedures for statistical inference could evolve into attractive computational assays providing estimates of ASD-specific disturbances in hierarchical Bayesian inference in individual patients.

Optimally, these assays should rest on paradigms, which are sufficiently appealing and independent of verbal instruction, such that they could be applied to young children. If successful, they could potentially replace the laborious gold standard ADOS/ADI-R diagnostics and provide an easier, faster diagnosis without reliance on trained specialists in expert institutions (*challenge i*). If such computerized assays are combined with response recording via eye tracking, they could become applicable in very young infants and potentially solve the problem of early recognition in high risk children at infant age (*challenge iii*). Similarly, they could be used for individuals without verbal skills at the severe end of the spectrum and help discriminate between ASD individuals with intellectual disability and those without spoken language but preserved intellectual and learning abilities (*challenge iv*).

Furthermore, such diagnostic assays could also solve the problem of recognizing autistic symptoms that are concealed by well-developed coping strategies in adolescence and adulthood (*challenge ii*). In this context, the Bayesian brain perspective has something to offer even before the development of novel diagnostic tests by suggesting a fundamentally novel theoretical explanation of the origin of autistic symptomatology. Its proposal of an abnormal balance in the precisions of sensory inputs and higher-order beliefs may facilitate a better understanding of the internal world of affected individuals, beyond the variety of observable manifestations. This novel explanatory model may support the education of clinicians with little previous exposure to individuals with ASD and help them grasp the potential range of clinical presentations. This alone may already help to reduce the number of unrecognized individuals with ASD (mainly in adult psychiatry).

One could speculate that, provided the research agenda outlined in this paper were successful, at some point in the future ASD might be redefined as “congenital perceptual inference disorder.” This redefinition on the basis of a generic pathomechanism might also affect other current diagnostic entities of adult psychiatry, such as schizoid or anankastic personality disorder. Given that their diagnostic criteria show major overlap with those of ASD, they could be regarded as foothills of the autism spectrum, with a possible relation to the same pathomechanism (146).

### Behavioral and Psychotherapy

The hierarchical Bayesian perspective may be useful for a better understanding of existing therapies. For example, the effectiveness of early intensive behavioral training to foster social interaction and speech development (18, 19) can be understood as a result of gently enforcing the child’s engagement in an interaction with the social environment, which is usually too noisy and dynamic for the affected child to be regarded as learnable and with which it, therefore, does not interact spontaneously. The core idea of the therapy is to reduce the complexity of social interactions to single, frequently repeated moments of interactions that slowly become interpretable (i.e., representable by a generative model) to the child and, therefore, manageable. From the Bayesian perspective, learning in ASD is more or less severely altered, but, nonetheless, possible throughout life, given optimal preconditions, such as little noise and dynamics in the exposed environment, and sufficient motivation for a high amount of repetitions. This view may trigger attempts to overcome the lack of behavioral therapies for severely affected adults (*challenge v*), as it suggests that these individuals should not be regarded as fundamentally limited in their capacity to learn. A continuation and adaptation of early behavioral training programs throughout life could slowly but steadily expand their scope of action and understanding. This approach is admittedly not without limitations. Actual intellectual disabilities – even if frequently overestimated – are certainly present, and a lack of motivation or resources are obvious obstacles, which cannot be overcome by theories alone.

Regarding autism-specific psychotherapy that goes beyond social skills training (*challenge vi*), two aspects can benefit from the explanatory appeal of the Bayesian brain perspective: psychoeducation, i.e., providing an explanation of the disorder, and psychotherapy in the strict sense, i.e., providing help in dealing and coping with the disorder. Concerning the former, and as observed ubiquitously across medicine, patients have a profound need to develop a concept of their disease and construct an explanation for their suffering. The perspective offered by the Bayesian brain theory can be useful in this regard, since it can be explained with reference to specific aspects of behavior and perception. It is the personal experience of the authors that this approach is useful for autistic patients and their relatives (but also their physicians) in order to establish a concept of their symptoms and suffering.

In treating autistic symptoms, two practical aspects take center stage: dealing with the perceived excessive complexity of everyday life and the fostering of developmental steps in the sense of expanding the scope of action. Generally, the principle “reduction of surprise by making the world predictable/understandable” may help patients deal with stress, sensory oversensitivity, and cognitive exhaustion by proactively seeking or creating predictable and controlled areas in both the personal and social domain. Complex social interactions can be rendered less perplexing or surprising by helping the patient to develop a generative model, by providing explicit explanations for the behavior of others and teaching them about possible reactions to chosen actions. In order to expand the scope of possible actions, the lack of spontaneous exploration and intuitive learning by generalization can be compensated by precise step-by-step instructions toward new actions that can be strengthened by excessive repetition. The generalization of such new abilities could be facilitated by explicit elaboration and memorization of the underlying abstract principle behind the behavior.

In the future, model-based estimates of individual abnormalities in hierarchical Bayesian inference could help facilitate the development of novel targeted psychotherapeutic interventions and subject-specific strategies for coping with cognitive deficits. For example, for patients where the relative overweighting of low-level vs. high-level precision (cf. Eq. 1) primarily derives from a deficit at high levels – for example, flat priors due to an overestimation of volatility – suitable interventions and coping strategies are likely to differ substantially from patients in whom overestimation of low-level sensory precision is the dominant problem. In the former case, inadequate higher cognitive levels of abstraction (flat top-down priors) might be sharpened by explicit teaching of abstract principles behind complex everyday processes, to buildup higher model levels without having to rely on the intuitive extraction of principles from exposure and imitation. In the latter case, efforts to reduce sensory overload by adaptions to the surrounding environment (at home, school, or work) seem more paramount.

The development of autism-specific psychotherapy of comorbidities (*challenge vii*) can likely profit from the novel explanatory perspective of ASD. Especially comorbidities, such as low mood or anxiety, can be understood as consequences rather than as incidental comorbidities with pathomechanisms that are completely independent from those of ASD. Their psychotherapy could also become more specific if patients understand these symptoms as a consequence of a fundamental perceptual problem, which causes chronic cognitive stress.

### Pharmacotherapy

Pharmacotherapy, in ASD, is presently mostly off-label (*challenge viii*) and, in the absence of predictive tests, necessarily relies on trial and error (*challenge ix*). The use of the approved dopamine receptor antagonist (risperidone) is of questionable benefit for cognition in ASD, as its antagonistic effects at dopamine receptors may interfere with precision-weighting of prediction errors, and thus updating of priors, in high-level regions of the cognitive hierarchy such as prefrontal cortex (91), which are critical for the development of abstract representations. Indeed, empirical studies demonstrate that core symptoms of ASD do not benefit from this medication (147). Perhaps not surprisingly, this purely symptomatic approach is rarely subjectively experienced as helpful by patients. Off-label use of pharmacotherapy mainly consists of stimulating different neuromodulatory systems that may be involved in precision-weighting of prediction errors: there is evidence of benefit by dopaminergic and noradrenergic stimulation with methylphenidate (25) and of purely noradrenergic stimulation with atomoxetine (148). The evidence for benefit of treatment with selective serotonin reuptake inhibitors is mixed (149). Several recent studies document first promising results for cholinergic stimulation (150) and oxytocin application (151). Future clinical studies in patient subpopulations, which are stratified by model-based indices of pathophysiology, might enable to select more effectively among available treatments; this could benefit in particular from computational functional neuroimaging analyses with potential sensitivity for alterations of neuromodulatory systems (118).

## Conclusion

Recently developed theories of ASD, which posit a fundamental perceptual abnormality due to an aberrant balance of precision estimates in hierarchical Bayesian learning, offer a novel and rich perspective on this spectrum disorder. In this paper, we have described the implications of this perspective for addressing central problems in the contemporary clinical management of ASD. We suggest that generative models of behavioral and functional neuroimaging data could play a key role in establishing novel objective diagnostic tests, which disambiguate patients characterized by different causes of the proposed perceptual aberration. Such models could become useful for selecting between existing pharmacological interventions and for developing novel behavioral/cognitive training programs. Close collaborations between clinicians and computational scientists will be essential for conducting the necessary translational studies.

## Author Contributions

HH, MS, and KS contributed to the conception, the drafting, and the revision of the manuscript. HH designed the figures.

## Conflict of Interest Statement

The authors declare that the research was conducted in the absence of any commercial or financial relationships that could be construed as a potential conflict of interest. The reviewer [NW] declared a shared affiliation, though no other collaboration, with the authors to the handling Editor, who ensured that the process nevertheless met the standards of a fair and objective review.

## Footnotes

^1^Stephan KE, Manjaly ZM, Weber L, Paliwal S, Mathys C, Gard T, et al. From dyshomeostasis to fatigue and depression – a Bayesian account of metacognition and self-efficacy. (In preparation).
